# Supplementary figures and images for: Evidence of Asexual Overwintering of Melampsora paradoxa and Mapping of Stem Rust Host Resistance in Salix
Source: Plants (Basel). 2022 Sep 13;11(18):2385. doi: 10.3390/plants11182385 (PMC9502555; doi:10.3390/plants11182385)

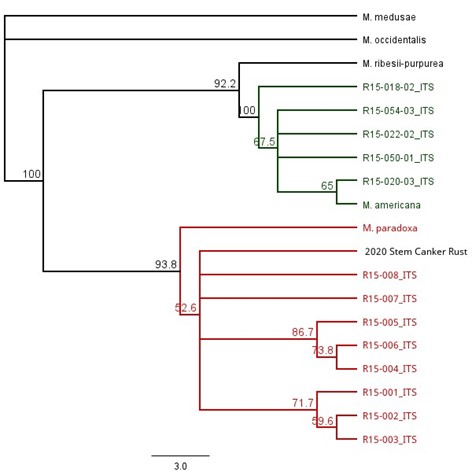

Supplement: Supplementary file 1 [file plants-11-02385-s001.zip › plants-1905263-supplementary.jpg]
